# Murine Left Atrium and Left Atrial Appendage Structure and Function: Echocardiographic and Morphologic Evaluation

**DOI:** 10.1371/journal.pone.0125541

**Published:** 2015-04-30

**Authors:** Francesca Colazzo, Laura Castiglioni, Luigi Sironi, Lucia Fontana, Elena Nobili, Matteo Franzosi, Uliano Guerrini

**Affiliations:** 1 Centro Cardiologico Monzino IRCCS, Milan, Italy; 2 Department of Pharmacological and Biomolecular Sciences, University of Milan, Milan, Italy; Univeristy of Glasgow, UNITED KINGDOM

## Abstract

Aim of this study was to provide an echocardiographic protocol for the description of the normal murine venous reservoir (atrium, appendage and pulmonary veins) and to investigate the possibility to use this approach to discriminate changes on left atrium (LA) and left atrial appendage (LAA) in a stress-induced model such us myocardial infarction. Global left ventricular function and the venous reservoir were assessed by a Vevo2100 in 20 female C57BL/6N. LA and LAA were also studied in 10 CD-1 and 10 FVB mice, whereas modifications investigated in 15 C57BL/6N subjected to coronary artery ligation. Left ventricle function was evaluated as well as pulsed Doppler mitral valve, pulmonary vein, and LAA velocities. From 2D view monoplane LA volumes were obtained and LAA long axis measured. Macroscopic inspection with casts and immunohistochemistry were performed. Results show that compared to humans, in C57BL/6N mice left atrium was disproportionately smaller (5.2±1.4μL) than the left ventricle (53±8μL) and connected through a duct by a large LAA and posteriorly to three pulmonary veins. The LA volume increased 2-fold during reservoir with two distinct phases, early and late divided by a short pause. LAA long axis (4.1±0.5mm) was almost 2 times longer than the LA. LAA flow volume together with LA volume reservoir account for about 36% of stroke volume and the rest was provided by conduit flow. Linear regressions showed that stroke volume was strongly influenced by LAA flow, LA early filling volume and left ventricle base descent. Moreover, we also report the ability to assess LA and LAA in other mice strains and discriminate size increase following myocardial infarction. In conclusion, we performed a complete characterization of murine left venous reservoir establishing an optimized protocol that can be used in both investigative and pharmacological studies requiring rapid and serial determination of cardiac structure and function.

## Introduction

Rodents are a powerful experimental model for a mechanistic understanding of normal cardiovascular function and to identify the molecular mechanisms underlying the pathological basis of cardiovascular diseases. Normal reference values have been established for murine left ventricle (LV) [1, 2] on the contrary, left atrium (LA) structure and function have never been evaluated. The LA acts as a venous reservoir during LV systole [3], enhances LV filling during early diastole, and contracts at end diastole to maximize LV end-diastolic volume and optimize stroke volume (SV). Extent of LA filling during the reservoir phase is an independent determinant of LV SV [3]; furthermore, LA size is both a prognostic determinant of cardiovascular risk and a marker of LV dysfunction [4].

Recent advances in imaging technology provide improved spatial and temporal resolution to visualize rodent myocardium, allowing the investigation of its smallest structures. High-frequency echocardiography is a non-invasive, accurate and reproducible tool for in vivo murine cardiovascular investigation [1, 2, 5–11]. The overall goal of the present study was to provide an optimized acquisition and analysis protocol for the assessment of anatomy and function of the murine left venous reservoir. Functional and geometric changes were also provided in a “stress-induced” model of myocardial infarction.

## Materials and Methods

### Animals

The procedures concerning animal care, surgery, and euthanasia were performed in accordance with national (D.L. n.116, G.U. suppl. 40, 18 February 1992) and International laws and policies (EEC Council Directive 86/609, OJL 358,1; 12 December 1987; NIH Guide for the Care and Use of Laboratory Animals, US National Research Council 1996) and approved and authorized by the National Ministry of Health-University of Milan Committee (Approval number 1242003- A 1371072003). Mice (Charles River Laboratories, Calco, Italy) were fed *ad libitum* with standard chow and water. All efforts were made to minimize suffering.

### Experimental Protocol

The left venous reservoir was assessed on twenty C57BL/6N 10-week-old female mice (18–20g) and on 10 CD-1 female mice (24–27g) and 10 FVB male mice (26–29g). Stress-induced changes were investigated on 15 C57BL/6N female mice subject to coronary artery ligation and compared with 15 sham-operated mice. Intergender differences were assessed by studing 10 male mice. Vevo 2100 system (VisualSonics, Toronto, Canada) equipped with a MS400 30-MHz linear array transducer were used for echocardiography. Induction of anesthesia was accomplished by exposing the mice to 2% isoflurane (Merial) in 100% Oxygen (5 mins) in an induction chamber. The mice were then placed in the supine position on a heated (37°C) platform with integrated electrode pads used to obtain electrographic signals and heart rate (HR). The mouse chest area was shaved and a warmed ultrasound gel applied to the thorax surface to optimize the visibility of the cardiac chambers. A nose cone was attached for continuous delivery of isoflurane (1% in 100% oxygen) titrated to maintain HR ≥450 beats per minute (bpm) during acquisition.

### Echocardiographic Examination

The Vevo MS400 30-MHz linear array transducer had an axial resolution of 50 μm and lateral resolution of 110 μm. Mean frame duration was 3.8 ms, and mean frames per cardiac cycle was 35.1±7.4. Respiration and ECG tracings were synchronised with imaging, and examinations lasted 30 min. Vevo software (VisualSonics, Toronto, Canada) was used for LV M-mode, 2D measurements, and pulsed Doppler velocities; Medimatic software (Medimatic, Genova, Italy) was used for the LA 2D measurements.

**Fig 1:**
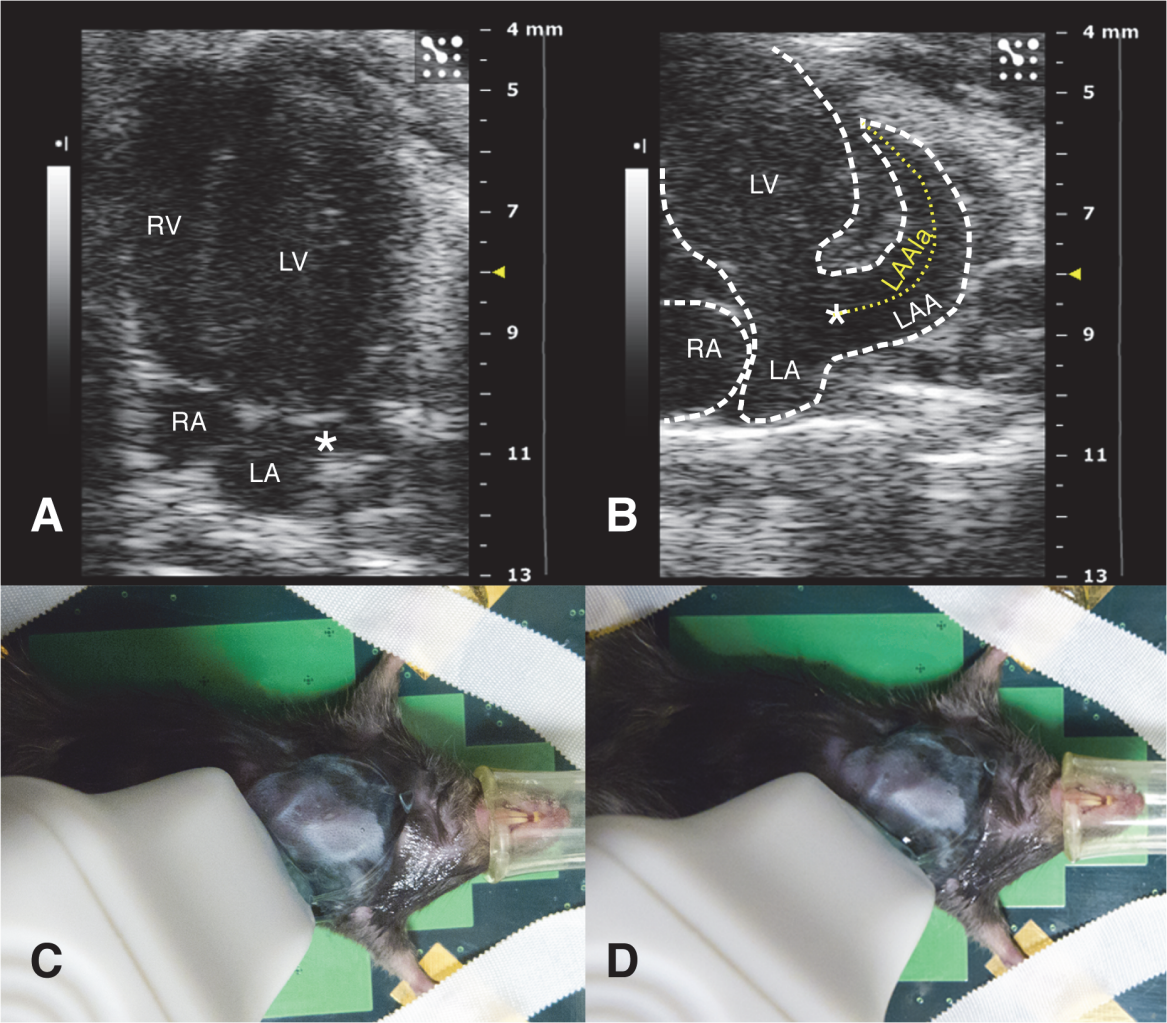
Standard and modified apical 4-chamber views. A) Standard apical 4-chamber view, optimized for the left atrium. B) Modified apical 4-chamber view to visualise the LAA and duct (*); the dotted line shows the measurement of the long axis C) Transducer position to obtain A. D) Transducer rotated counter-clockwise starting from C to obtain B. *: LA-LAA duct; LA: left atrium; LAA: left atrial appendage; LAAla: LAA long axis; LV: left ventricle; RA: right atrium; LV: left ventricle; RV: right ventricle.

**Fig 2:**
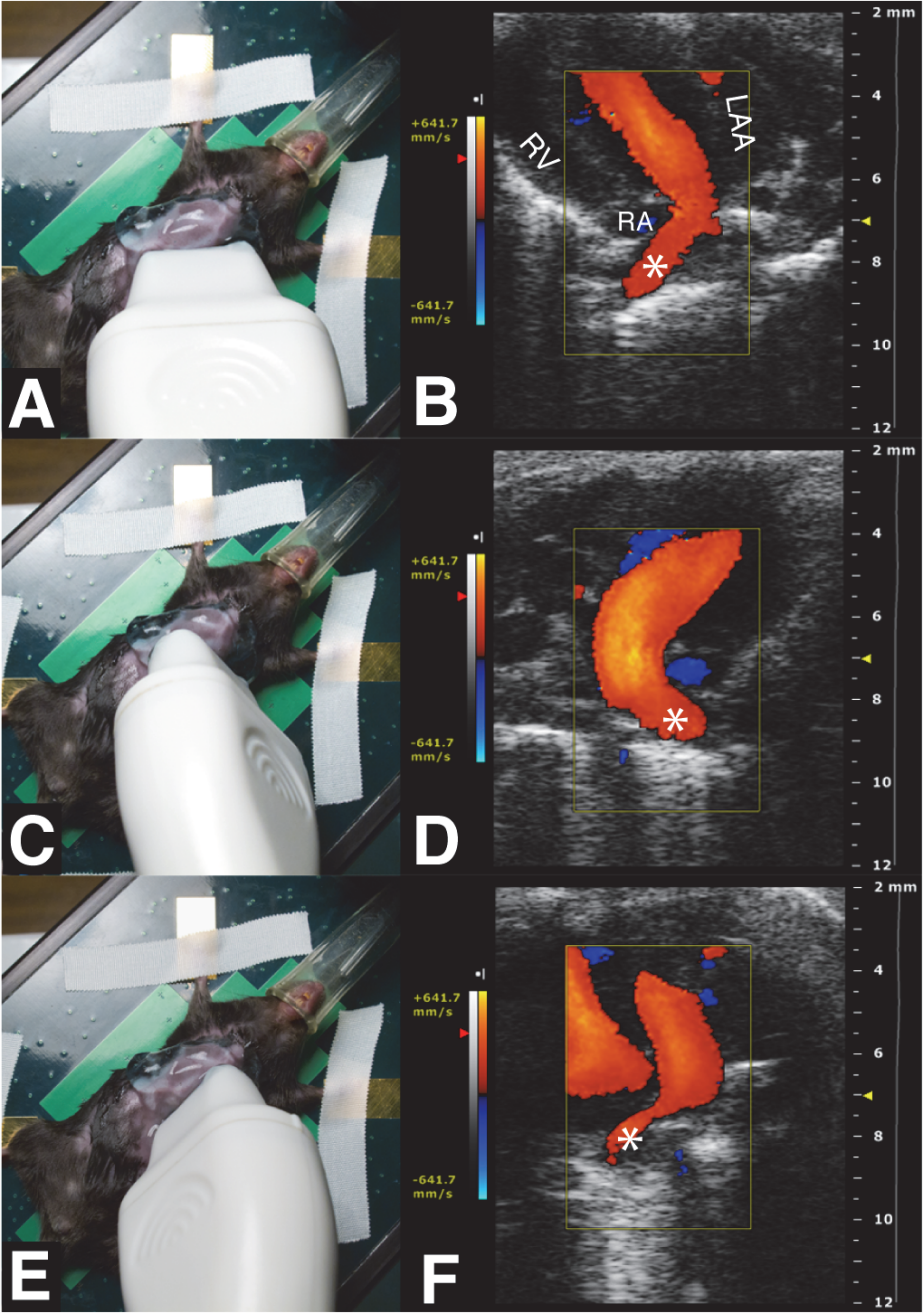
Color Doppler and anatomy of the three pulmonary veins. A) Modified apical 4-chamber view to visualise the right superior PV. B) Color Doppler LA inflow from the right superior PV. C) Transducer rotated clockwise to visualise left PV. D) Color Doppler LA inflow from the left PV. E) Transducer rotated counter-clockwise to visualise right inferior PV. F) Color Doppler LA inflow from right inferior PV. *: PVs; LAA: left atrial appendage; RA: right atrium; RV: right ventricle.

Based on a previous pilot study, mice were imaged in: 1) the left parasternal long axis view (2D LV long axis); 2) the parasternal short axis view (M-mode and 2D LV dimensions and systolic function); 3) the standard apical 4-chamber view optimized for the LA cavity (2D LA dimensions and function; mitral valve (MV) annulus dimensions and mitral pulsed Doppler flow velocity) (Fig 1A and 1C); and 4) modified apical 4-chamber views: a) a counter-clockwise rotation to optimize LAA imaging (2D dimensions and pulsed Doppler flow velocity), the LA-LAA duct, and a right pulmonary vein (PV) (Fig 1B and 1D); and b) clock- and counter-clockwise rotations in order to image additional PVs (Fig 2A–2F). Pulsed Doppler PV flow sampling was limited to the former view.

### ECG Measurements

The R-R intervals were measured, and the Q wave used as a time reference for pulsed Doppler blood flow velocities.

### Echocardiographic Measurements

#### Left ventricle

The end-diastolic and end-systolic LV areas (three short axis views located at the base, mid-papillary level and apex) were measured, and LV end-diastolic, end-systolic volumes, stroke volume, ejection fraction and cardiac output were calculated using the Simpson’s rule. M-mode LV short axis end-diastolic and end-systolic diameters, and posterior and anterior wall thicknesses, were measured, short axis fractional shortening, and LV mass (Troy formula) were calculated. The end-diastolic and end-systolic LV long axis were measured and long axis systolic shortening was calculated. Isovolumic contraction (IVCT) and relaxation (IVRT) and LV ejection times were measured on pulsed Doppler recordings of simultaneous LV outflow and transmitral flow and used to calculate the myocardial performance index, an indicator of global function, as [(IVCT + IVRT) / LV ejection time].

**Fig 3:**
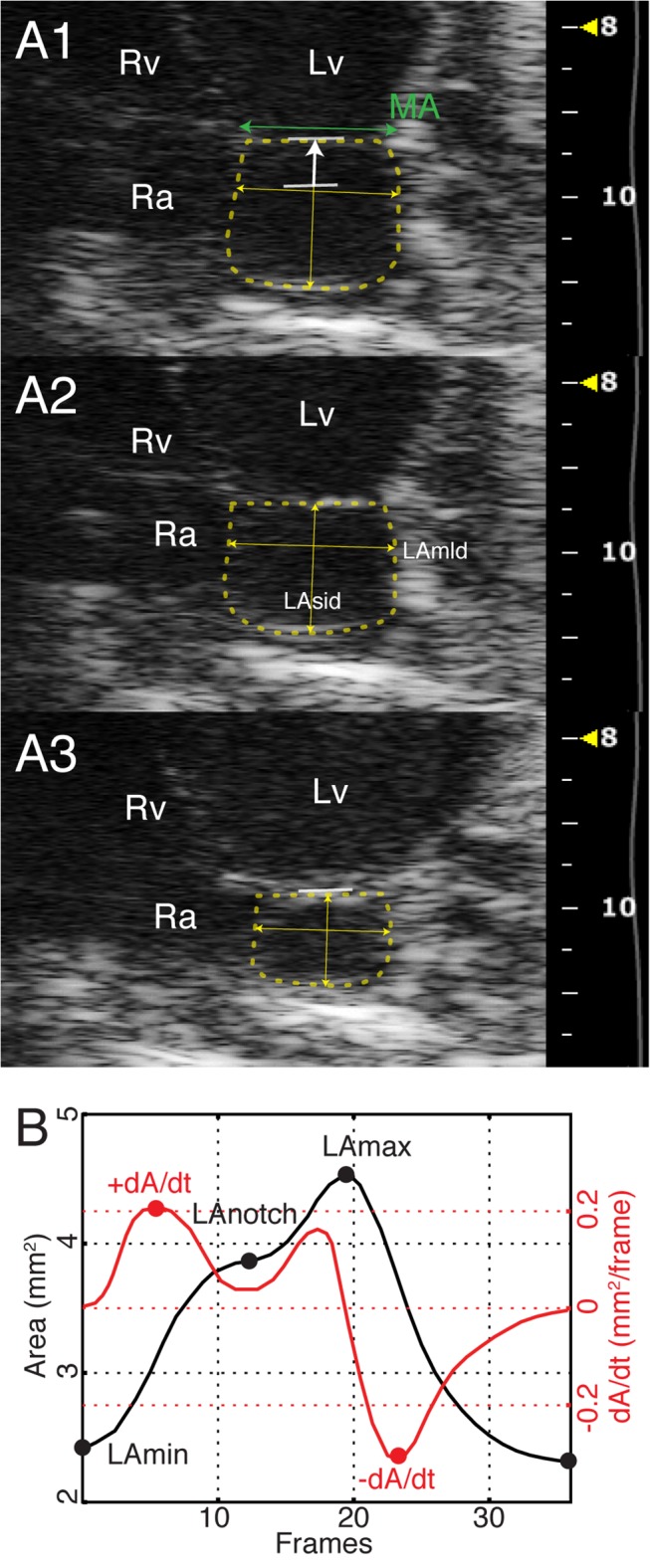
Left atrial 2D area, diameters and function curve. A) LA area planimetry in maximum (A1), “notch” (A2) and minimum (A3) dimensions; apical 4-chamber view. White arrow: systolic displacement of the mid-point of the mitral annulus (LV base descent). B) LA function curve in normal mouse. LA: left atrial; LAmld: LA medio-lateral (transverse) diameter; LAmax: maximum LA area; LAmin: minimum LA area; LAnotch: intermediate LA area at « notch »; LAsid: LA supero-inferior (longitudinal) diameter; MA mitral annulus; +dA/dt: peak positive reservoir LA area change;-dA/dt: peak negative emptying LA area change.

LV base descent was masured as mitral valve (MV) mid point displacement (Fig 3A1), MV peak velocity and total velocity-time integral were measured.

#### Left atrium

In the apical 4-chamber view, on the basis of frame-by-frame inspection, three frames were identified and their timing recorded: minimum (LAmin, one frame before LA expansion), maximum (LAmax, one frame before mitral valve opening), and an intermediate frame when there was a pause in the increase in LA dimension, defined as LA “notch” (LAnotch) (Fig 3A). Monoplane minimum, notch and maximum volumes (Simpson’s rule) and absolute volume changes during early (LAnotch – LAmin), late (LAmax – LAnotch) and total (LAmax – LAmin) LA reservoir filling were calculated. Minimum and maximum longitudinal (supero-inferior, from the mid point of the mitral annulus to the superior wall) and transverse diameters (medio-lateral, from the interatrial septum to the LA lateral wall, using the upper border of the LA-LAA duct as a marker) were measured (Fig 3A). The early, late, and total reservoir filling durations were calculated and normalized on the RR period.

LA function curve was built using frame-by-frame planimetry; peak systolic area increase (reservoir, +dA/dt), and peak diastolic decrease (contraction,-dA/dt) were calculated. The ratio between supero-infero and medio-lateral diameters at minimum and maximum LA dimensions was calculated.

#### Left atrial appendage

**Fig 4:**
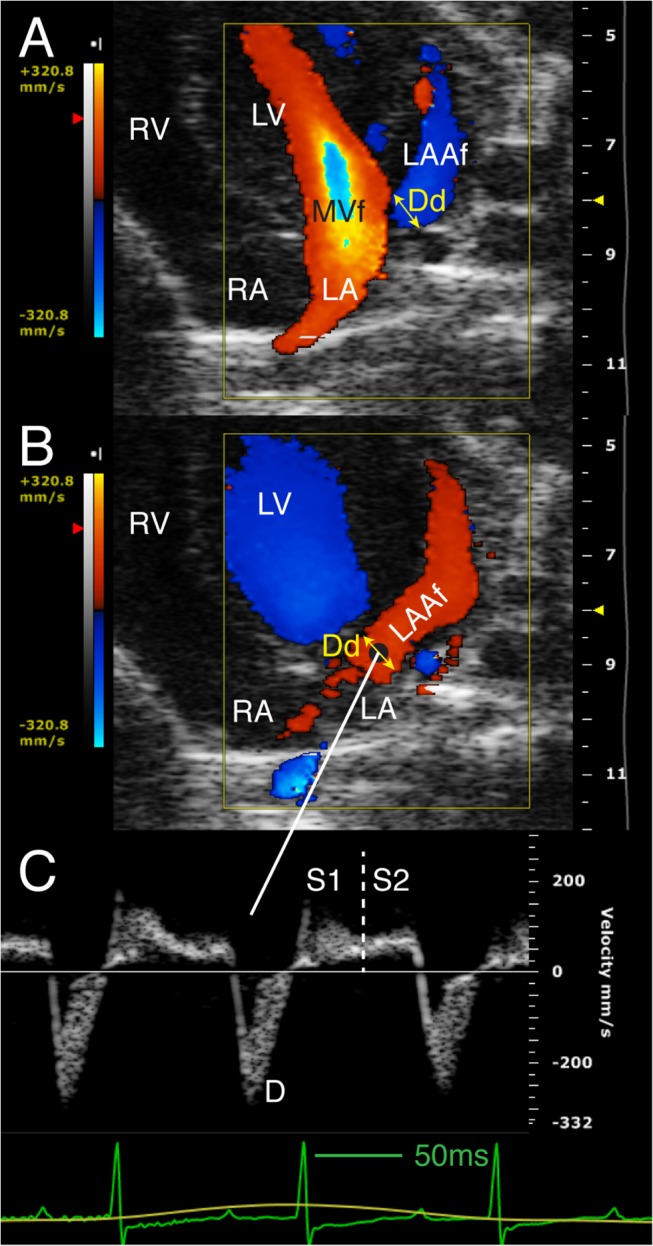
Left atrial appendage color and pulsed Doppler flow velocities. Modified apical 4-chamber view. Color (A, B) and pulsed Doppler LAA flow velocity (C), and measurement of LA-LAA duct diameter. A) Diastolic frame. B) Systolic frame. Green line on the ECG shows a 50 ms duration. Dd: LA-LAA duct diameter; LA: left atrium; LAA: left atrial appendage; LAAf: LAA flow; LV: left ventricle; MVf: mitral valve flow; RA: right atrium; RV: right ventricle; S1: early systolic inflow wave; S2: late systolic inflow wave; D: diastolic outflow wave.

The LAA maximum long axis (mid-line curve between the LAA apex and duct during LV end-systole) (Fig 1B), the minimum and the maximum duct diameters were measured (Fig 4A and 4B). Mean duct area was calculated assuming a circular orifice and duct diameter fractional shortening as [(maximum – minimum) / maximum × 100]. Based on the pilot study results, three waves for the trans-ductal LAA flow pattern were identified: two positive filling waves (early S1 and late S2) and a negative diastolic outflow wave (D) (Fig 4C). Duration, peak velocity and the velocity-time integral of each wave were measured. Appendage stroke volume (LAA SV) was estimated as the product of mean duct area by the maximum between the total inflow integral (S1+S2) and total outflow integral D.

#### Pulmonary vein

**Fig 5:**
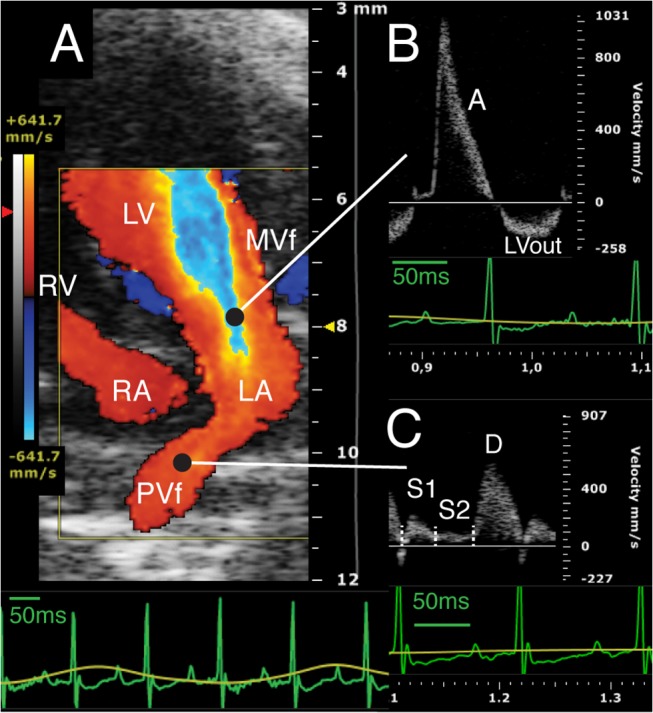
Mitral valve and pulmonary vein flow velocities: color and pulsed Doppler. A) Color Doppler flow velocity, standard 4-chamber view, diastolic frame. B) Pulsed Doppler MVf velocity profile. C) Pulsed Doppler PVf velocity profile. Green line on the ECG shows a 50 ms duration. A: MV wave; D: PV diastolic wave; LA: left atrium; LV: left ventricle; LVout left ventricle outflow; MVf: mitral valve flow; PVd right superior pulmonary vein diameter; PVf: right superior pulmonary venous flow; RA: right atrium; RV: right ventricle; S1: PVf early systolic wave; S2: PVf late systolic wave.

The flow pattern in the pilot study showed two forward LA filling waves (early S1 and late S2), a single forward diastolic wave (D) often followed by a small pulmonary venous atrial reversed flow wave (Fig 5A and 5C). The duration, peak velocity and velocity-time integral of each wave were measured, and, total systolic and total PV integral calculated.

#### Atrial Reservoir and Conduit Flow

Total reservoir volume change was calculated as: total 2D LA reservoir filling + LAA pulsed Doppler reservoir inflow. Total conduit flow was calculated as: LV SV – total reservoir volume change.

### Echocardiographic Variability

We evaluated interobserver variability of selected indices by comparing echocardiographic data obtained by 2 independent observers within 1 session of echocardiographic acquisition from 10 mice and intraobserver variability results obtained by the same observer within one session on different days. For intersession variability, indices from the same 10 mice were recorded twice by the same observer with a time interval of 1 week. Interobserver and intraobserver variability within one session and intersession variability with the same observer were expressed as the percent discrepancy between two measurements (i.e the absolute value of the difference between the two measurements divided by the mean of the two, expressed as a percentage) [12].

### Surgery

Twenty C57BL/6N 10-week-old female mice were anesthetized and visualized by echocardiography at baseline, then anestetized by intraperitoneal injection of a mixture of ketamine hydrochloride (75 mg/kg) and medetomidine hydrochloride (1 mg/kg), intubated and ventilated with positive airway pressure. After thoracotomy, myocardial infarction was induced by permanent ligation of the left anterior descending (LAD) coronary artery as previously reported [13]. A group of mice (n = 15) undergoing thoracotomy without ligation of the LAD (sham) was used as controls. After surgery atipamezolo (0.05 mg/kg) was administered to encourage animal awakeninig, then the animals were extubated and monitored. For MI-mice, LV echocardiograpy was peformed at 24 hours after surgery and only mice with ejection fraction ≤ 45% (n = 15) were included in the study. After 1 and 4 weeks comprehensive LV and LA echocardiography was performed in both groups.

### Vascular Casts and Tissue Preparation

After echocardiography, mice were sacrified by intraperitoneal injection with ketamine hydrochloride (75 mg/kg) and medetomidine hydrochloride (1 mg/kg). For macroscopic inspection, hearts (n = 10) were perfused with a silicone elastomer (1 ml/min) through the cannulated abdominal aorta to build a vascular cast. For histology and immunofluorescence, hearts (n = 10) were arrested in diastole with CdCl_2_ then retrogradely perfused for 10 mins each with 0.01M phosphate saline buffer (PBS) and 4% (vol/vol) phosphate-buffered formalin, then postfixed in 4% phosphate-buffered formalin for 24 hrs and embedded in Paraplast.

#### Histology

C57BL/6N mice hearts were sectioned coronally (8 μm), deparaffinized and rehydrated. Haematoxylin-eosin staining was performed for morphologic/topologic evaluation, and Masson’s Trichrome staining for collagen detection. Slides were viewed under a light microscope (Zeiss Axioskop) and images were acquired using a digital camera with a 1:1 macro-lens.

#### Immunofluorescence

Heart sections, de-waxed/rehydrated, were subject to heat-induced antigen retrieval in sodium citrate buffer (10mM sodium citrate, 0.05% Tween 20, pH 6.0) and then incubated for 45 mins at room temperature in 10% normal goat serum (Dako) in PBS 0.01M and 0.1% Triton X-100. Primary antibodies were applied overnight at 4°C and secondary antibodies for 2 hrs at room temperature. All antibodies were prepared in PBS and 0.1% Triton X-100. For nuclear staining, the sections were incubated with Hoechst 33258 (2.5 g/ml; Invitrogen) in PBS for 15 min, before slide covering and fluorescence miscroscope (200M; Zeiss) observation. For cardiomyocytes, smooth muscle cells and cardiac fibroblasts evaluation, sections were incubated respectively with mouse monoclonal antibody anti-α-sarcomeric actin (1:800), α-smooth muscle actin (1:600), or vimentin (1:1500) (all Sigma-Aldrich). For endothelial cells evaluation, endogenous peroxidase activity was blocked in 3% H_2_O_2_ in methyl alcohol/PBS 50/50 for 20 min, and then the sections incubated overnight at 4°C with isolectin B4 peroxidase (1:100; Sigma-Aldrich). Appropriate secondary antibodies (Alexa 555 goat anti-mouse IgG [1:600, Invitrogen], Alexa 546 goat anti-mouse IgM [1:600, Invitrogen], and fluorescein-conjugated goat anti-horseradish peroxidase [1:100 Jackson ImmunoResearch]) were applied. The staining was semi-quantitatively assessed by two blinded operators, and the average of the two analyses was reported.

### Statistical Analysis

All echocardiographic data are the average of three measurements at end-expiration. Gender differences were compared using unpaired t test, while paired t test was used for baseline versus MI comparisons. Univariate linear regression was performed on selected indices. To obtain a multivariate linear regression of LV SV all the significant univariate predictors of LV SV were combined to define the maximal model that was then simplified by backward elimination to the minimal adequate model of LV SV. All statistical analyses were performed using R 2.15 (R Core Team; Vienna, Austria), and p <0.05 was considered significant. All data are presented as mean ± 1 SD.

## Results

No significant gender related differences were found therefore we report only female values. HR was 519±34. LV echocardiographic measurements are shown in Table 1.

**Table 1:** Left ventricle: volumes, anatomy and function.

End-diastolic Volume	53 ± 8 μL
End-systolic Volume	13.8 ± 4 μL
Stroke Volume	39.1 ± 7 μL
Cardiac Output	19.8 ± 4 ml/min
Ejection Fraction	74 ± 7%
End-diastolic diameter	3.7 ± 0.3 mm
End-systolic diameter	2.1 ± 0.3 mm
Fractional shortening	44 ± 6%
Anterior wall, diastole	0.75 ± 0.2 mm
Anterior wall, systole	1.38 ± 0.16 mm
Posterior wall, diastole	0.83 ± 0.14 mm
Posterior wall, systole	1.25 ± 0.17 mm
Mass	107 ± 19 mg
Long axis, diastole	7.3 ± 0.5 mm
Long axis, systole	5.9 ± 0.4 mm
Long axis shortening	19 ± 4%
Base descent	0.63 ± 0.13 mm
Isovolumic contraction duration	9 ± 4% (of QQ)
Ejection duration	40 ± 3% (of QQ)
Isovolumic relaxation duration	11 ± 4% (of QQ)
Myocardial performance index	0.52 ± 0.17

Values are mean±SD.

### Left Atrial Anatomy and Function

LA was apically imaged in all mice as a small, almost spherical, structure communicating laterally through a short duct, originating in the LA lateral wall, with a much larger, crescent-shaped, LAA (Figs 1 and 3). The maximum LA volume was 10% of the LV end-diastolic volume as indicated from data in Tables 2 and 1. The LA supero-inferior to medio-lateral diameter ratio increased at end-reservoir (maximum *vs* minimum: p<0.001). Early filling is prevalent: in particular its duration and volume increase doubled that of late filling. (Fig 3; Table 2). In all mice the atrial function curve showed a single emptying slope (Fig 3B) after the ECG P wave. Atrial 2D reservoir filling accounted for 7–8% of LV SV.

**Table 2:** Left atrium: volumes, anatomy and function.

Early reservoir filling duration	36±5% (of RR)
Late reservoir filling duration	18±4% (of RR)
Total reservoir filling duration	54±5% (of RR)
Volume, min	2.3±0.7 μL
Volume, notch	4.2±1.3 μL
Volume, max	5.2±1.4 μL
Early reservoir volume increase	2.0±0.8 μL
Late reservoir volume increase	1.0±0.4 μL
Total reservoir volume increase	3.0±0.9 μL
MLD, min	1.6±0.2 mm
SID, min	1.4±0.2 mm
MLD, max	2.0±0.2 mm
SID, max	2.1±0.2 mm
SID/MLD, max	1.05±0.13
SID/MLD, min	0.89±0.14
Peak systolic area increase dA/dt	26±9 mm^2^/s
Peak diastolic area decrease-dA/dt	-45±17 mm^2^/s

MLD: medio-lateral diameter, SID: supero-inferior diameter. Values are mean±SD.

### Left Atrial Appendage Anatomy and Flow Profile

LAA could be visualized and measured in all mice. Long axis (4.15±0.5mm) was almost 2 times longer than the LA (2±0.2mm). Its dimension and duct diameter increased during filling and decreased during emptying. The early inflow wave duration, peak velocity, and integral were all greater than those of late inflow wave (*p*<0.001 for all). LAA SV (11.8±4μl) was about four times the LA total reservoir volume increase (3±0.9 μl). Total (LA+LAA) venous reservoir was 14.7±4.5 μl and conduit volume was 24.4±4.4 μl. Results are presented in Table 3.

**Table 3:** Left atrial appendage: anatomy and pulsed Doppler flow profile.

Early filling wave, duration	36 ± 3% (of RR)
Early filling wave, peak velocity	283 ± 100 mm/s
Early filling wave, integral	8.1 ± 2 mm
Late filling wave, duration	28 ± 3% (of RR)
Late filling wave, peak velocity	168 ± 40 mm/s
Late filling wave, integral	4.8 ± 1.3 mm
Total filling wave, integral	12.9 ± 3 mm
Emptying wave, duration	33 ± 4% (of RR)
Emptying wave, peak velocity	430 ± 130 mm/s
Emptying wave, integral	10.5 ± 3 mm
LAA long axis, maximum	4.15 ± 0.5 mm
LAA / LA long axis, maximum	2 ± 0.4
Duct diameter, minimum	0.7 ± 0.12 mm
Duct diameter, maximum	1.4 ± 0.17 mm
Duct diameter, fractional shortening	49 ± 6%
LAA stroke volume	11.8 ± 4 μL

LA: left atrium, LAA: left atrium appendage. Values are mean±SD.

### Pulmonary Venous Anatomy and Flow Profile

Three different PVs entering the LA cavity at different angles were imaged: a right superior (next to the LA septum and opposite to the LAA duct) (Fig 2D), a right inferior and a left PV (Fig 2E and 2F). Doppler PV data are presented in Table 4. Early systolic wave peak velocity and integral were greater than late (both *p*<0.05).

**Table 4:** Right superior pulmonary vein pulsed Doppler flow profile.

Early systolic wave, duration	26 ± 3% (of RR)
Early systolic wave, peak velocity	190 ± 60 mm/s
Early systolic wave, integral	5.3 ± 1.7 mm
Late systolic wave, duration	28 ± 4% (of RR)
Late systolic wave, peak velocity	145 ± 60 mm/s
Late systolic wave, integral	2.6 ± 1.5 mm
Total systolic wave, integral	7.9 ± 2.6 mm
Diastolic wave, duration	42 ± 7% (of RR)
Diastolic wave, peak velocity	600 ± 120 mm/s
Diastolic wave, integral	18.7 ± 4.6 mm
Total PV integral	26.6 ± 5.5 mm

PV: pulmnunary vein. Values are mean±SD.

### Mitral Annulus and Valve Flow Profile

A single pulsed Doppler wave was detected in all mice and, as it followed the ECG P wave, it was then labeled as A wave (Fig 5B). A conventional pattern with both E and A waves was observed by increasing anesthesia (>1,5% isoflurane) until the HR decreased under about 400 bpm (data not show). Peak A wave was 880±120cm/s and total velocity-time integral was 21.7±4.3mm. In 2D four-chamber view annulus size and LV base descent were also measured (respectively 1.8±0.3mm and 0.63±0.13mm).

### Determinants of Stroke Volume

At univariate analysis the strongest correlation was found between LV SV and LAA SV while at multivariate analysis the main independent determinants of LV SV were LAA SV and LV base descent. Significant multivariate and univariate linear regression are reported in Table 5.

**Table 5:** Multivariate and univariate linear regression analysis.

Dependent	Adj. R^2^	p	Predictor	B	SE	p	
LV SV	0.70	<0.001	LAA SV	1.04	0.25		<0.001
			LV BD	22.8	7		0.008
			Intercept	12.2	5		0.02
LV SV	0.55	<0.001	LAA SV	1.26	0.25		<0.001
			Intercept	24.1	3.2		<0.001
LV SV	0.41	0.006	LA Vres early	5.9	1.8		0.006
			Intercept	26.7	3.8		<0.001
LV SV	0.37	0.003	LV BD	35	10		0.003
			Intercept	16.9	6.5		0.02
LV SV	0.36	0.003	PV integral	0.8	0.2		0.003
			Intercept	17.8	6.3		0.01
LV SV	0.18	0.03	LA Vres	3.9	1.7		0.03
			Intercept	27.6	5.2		<0.001
LAA SV	0.41	0.0013	PV systolic integral	1.1	0.3		0.0013
			Intercept	3.2	2.3		0.19
LAA SV	0.22	0.02	LAA length	3.9	1.5		0.02
			Intercept	-4.5	6.4		0.49
LA Vres	0.20	0.03	+dA/dt	0.05	0.02		0.03
			Intercept	1.7	.6		0.006

BD: base descent, LA: left atrium, LAA: left atrium appendage, LV: left ventricle, PV: pulmonary vein, SV: stroke volume, Vres: volume reservoir, +dA/dt: peak systolic area increase.

### Macroscopic Anatomy and Histology

**Fig 6:**
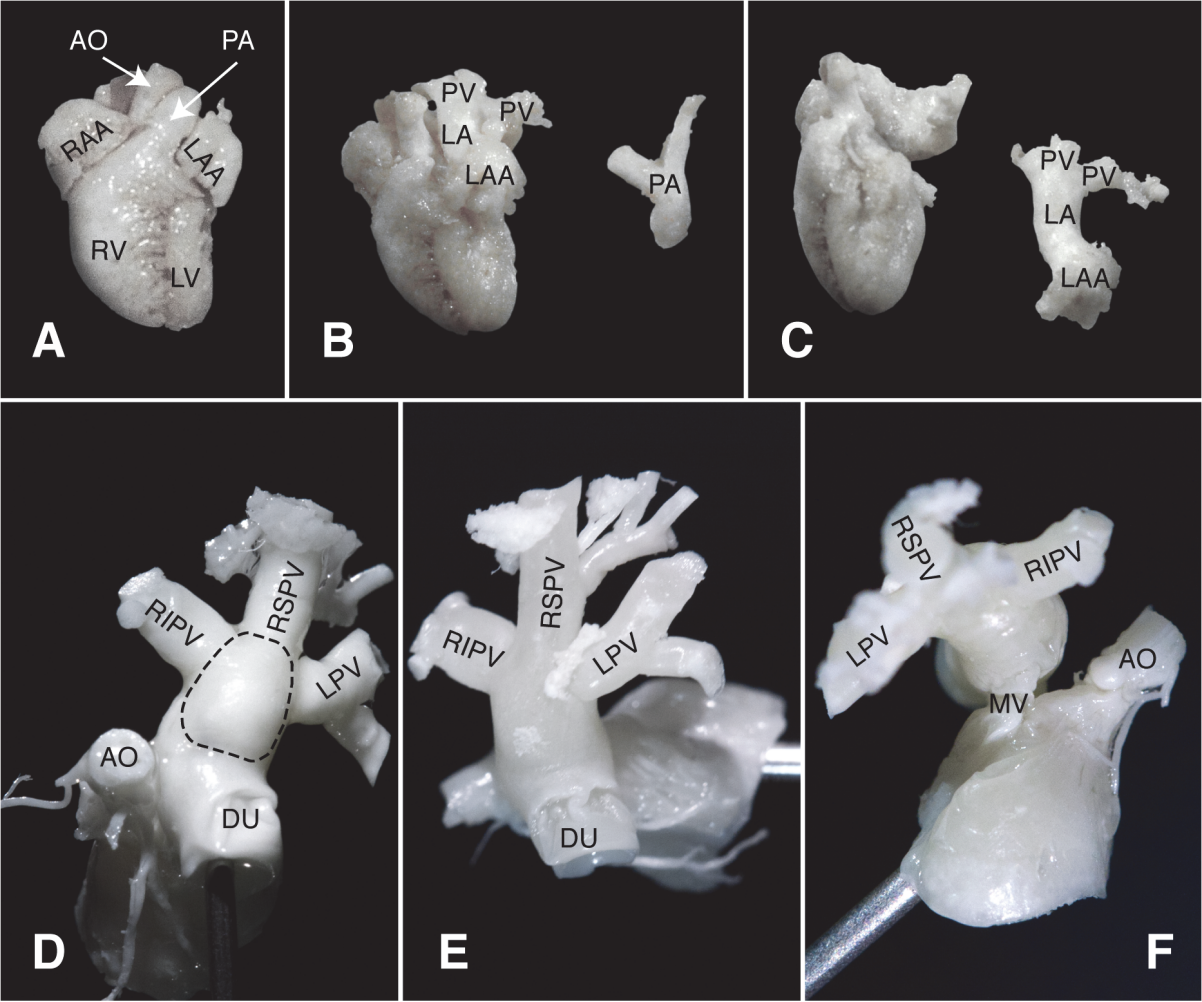
Heart and left venous reservoir resin casts. A) Whole heart, front view. B) As in A, after the removal of the main pulmonary artery and bifurcation. C) As in B, after the removal of the left venous reservoir (LA, LAA and PV). D) Isolated left venous reservoir, frontal-cranial view with LA (superior wall; the dotted line delimits the LA cavity), AO with coronary arteries and RIPV, RSPV and LPV. E) Isolated left venous reservoir, cranial view with LA (superior wall), RIPV, RSPV and LPV. F) Isolated left venous reservoir, inferior view from the right with LA (inferior wall), RIPV, RSPV and LPV, and MV connection to the LV. Shown needle gauge: 21. AO: aortic root; DU: LA-LAA duct; LA: left atrium; LAA: LA appendage; LPV: left PV; LV: left ventricle; MV: mitral valve; PA: pulmonary artery; PV: pulmonary veins; RIPV: right inferior PV; RSPV: right superior PV; RV: right ventricle.

**Fig 7:**
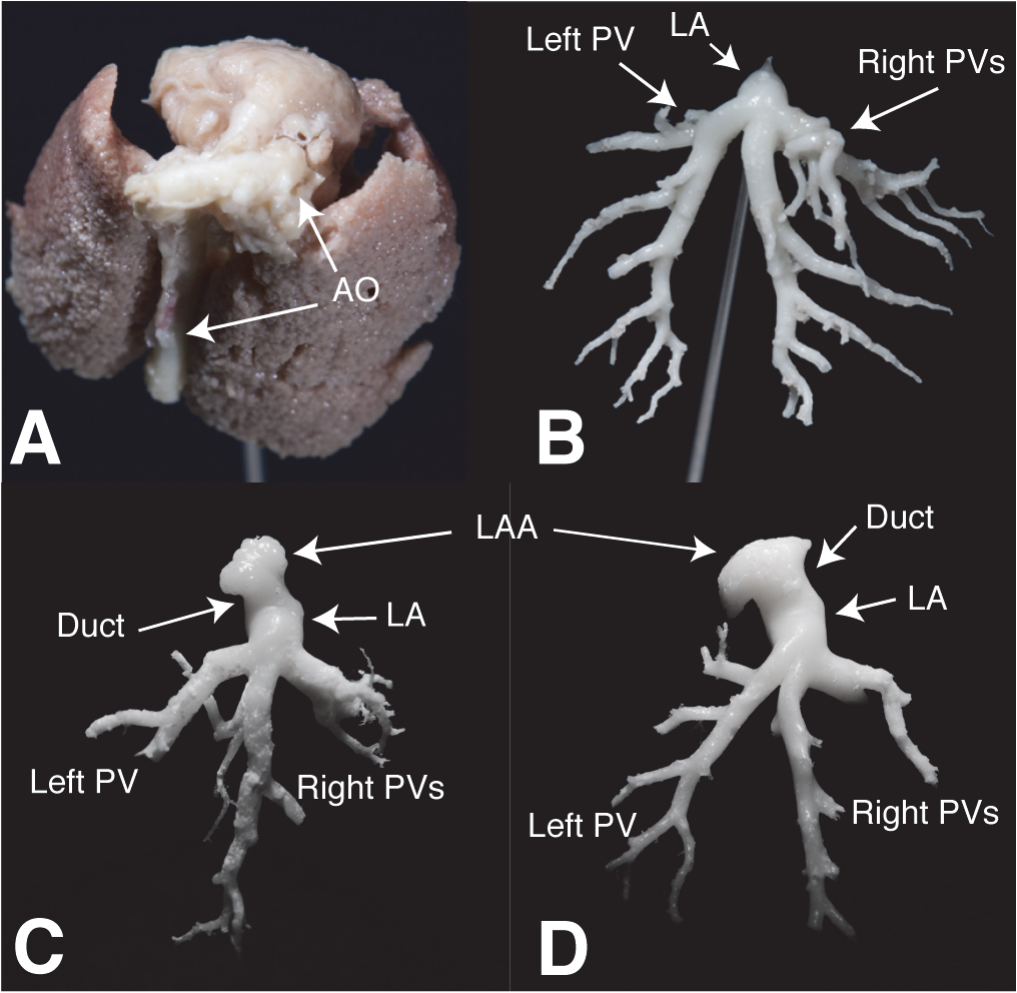
Vascular Cast. A) Representative cast of the C57/BL6 mouse venous reservoir *in situ* with lungs (showing the dominance of the right lung) and AO. B) PV vascular cast of C57/BL6 mouse showing the prevalence of right venous outflow (confluence in two PVs) over the left (one PV). CD-1 (C) and FVB (D) PV vascular cast. Shown needle gauge: 21. AO: aortic arch; PV: pulmonary veins; LA: left atrium; LAA: left atrial appendage.

Resin casts showed that the trabeculated LAA could be identified frontally, lying on the LV base. Removing the pulmonary artery, LA and its connections were visualized: antero-laterally with LAA, inferiorly with the LV through the mitral valve, and postero-medially with the three PVs (60° angle between each PV) (Fig 6). The two right PVs come from the dominant bilobate right lung (Fig 7A and 7B).

**Fig 8:**
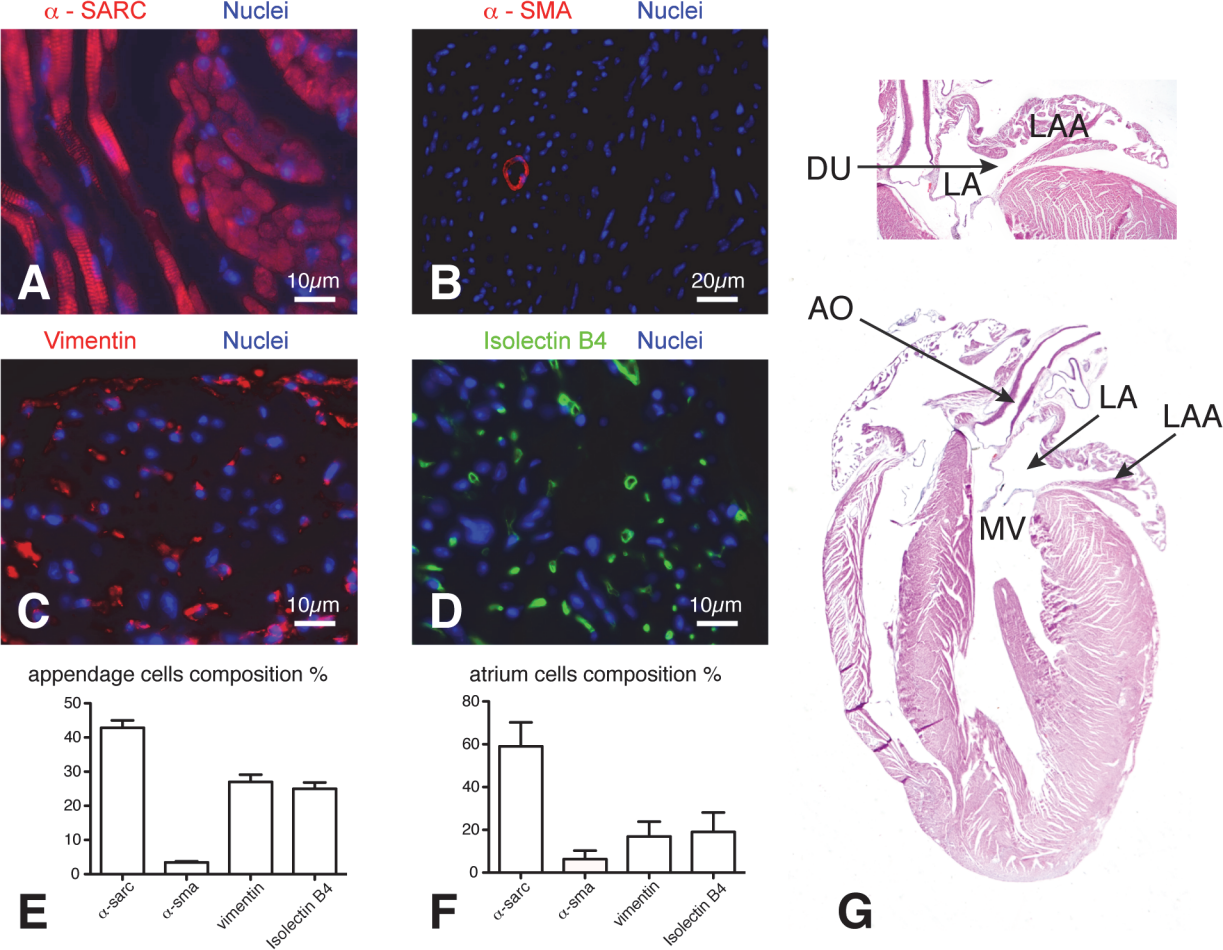
LA and LAA histology and cellular composition. The LA and LAA bodies consisted of two thin layers of cardiomyocytes (A) crossing each other, and cardiac fibroblasts (C). Both structures exhibited a dense capillary network (D) and few α-smooth muscle actin positive cells (B). Graphs E and F illustrate cells percentage within LAA and LA. In G, murine heart coronal 4-chamber section. α -sarc: alpha-sarcomeric actin; α -sma: alpha-smooth muscle actin; AO: aorta; DU: LA-LAA duct; LA: left atrium; LAA: LA appendage; MV: mitral valve.

Both LA and LAA consisted of cardiac fibroblasts (respectively 16±6.9% *vs*. 28±7.4% vimentin) and two layers of cardiomyocytes (58±10% *vs* 44±5.8% α-sarcomeric actin) crossing each other. Both structures showed a dense capillary network (19±8.9% *vs* 25±6% isolectin-B4) and few well-organised α-smooth muscle actin-positive cells (5.7±4% *vs* 3.5±1%) (Fig 8). Only mitral and aortic valve leaflets stained positively at Masson trichrome (not shown).

### Echocardiographic and Macroscopic Evaluation of CD-1 and FVB Mice

Cardiac examination time was around 30–40 min, in all animals. Mean body weight was 26.13±1.59 g and 28±1.3 g for CD-1 and FVB mice respectively, whereas heart rate was 493±28 bpm and 541±29 bpm. Technically adequate images could be obtained in all animals. Left atrium was apically imaged with its connection with the appendage. Three PVs entering in LA were immaged in both strains. These data were also confirmed by gross macroscopic evaluation of the CD-1 and FVB hearts resin casts (Fig 7C and 7D). All echocardiographic measurements performed are summarized in Tables 6 and 7.

**Table 6:** Selected echocardiographic indices in CD-1 mice.

**Left Ventricle**	
End-diastolic Volume	41 ± 5.75 μL
End-systolic Volume	9.2 ± 2.7 μL
Stroke Volume	31.9 ± 5 μL
Ejection Fraction	77 ± 6%
Fractional shortening	47 ± 7%
Myocardial performance index	0.79 ± 0.16
Mass	101 ± 19 mg
**Left Atrium**	
Volume, min	3.08 ± 0.9 μL
Volume, notch	5 ± 1 μL
Volume, max	6.4 ± 1.2 μL
Early reservoir volume increase	2 ± 0.6 μL
Late reservoir volume increase	1.3 ± 0.7 μL
Total reservoir volume increase	3.3 ± 0.6 μL
MLD, min	1.6 ± 0.1 mm
SID, min	1.7 ± 0.2 mm
MLD, max	2.1 ± 0.1 mm
SID, max	2.2 ± 0.2 mm
**Left Atrial Appendage**	
LAA long axis, maximum	3.8 ± 0.3 mm
Duct diameter, minimum	0.75 ± 0.1 mm
Duct diameter, maximum	1.32 ± 0.13 mm
Duct diameter, fractional shortening	43 ± 4%
LAA stroke volume	8 ± 1.6 μL
**Right Superior Pulmonary Vein Flow Profile**	
Early systolic wave, peak velocity	151 ± 38 mm/s
Early systolic wave, integral	5.3 ± 1.5 mm
Late systolic wave, peak velocity	100 ± 37 mm/s
Late systolic wave, integral	3 ± 1.2 mm
Diastolic wave, peak velocity	562 ± 130 mm/s
Diastolic wave, integral	19.6 ± 3.7 mm
Total PV integral	26 ± 5 mm

MLD: medio-lateral diameter, SID: supero-inferior diameter, PV: pulmonary veins.

Values are mean±SD.

**Table 7:** Selected echocardiographic indices in FVB mice.

**Left Ventricle**	
End-diastolic Volume	49 ± 6.4 μL
End-systolic Volume	12 ± 3.1 μL
Stroke Volume	36 ± 4.5 μL
Ejection Fraction	74.8 ± 4.3%
Fractional shortening	36 ± 4.4%
Myocardial performance index	0.65 ± 0.2
Mass	101 ± 19 mg
**Left Atrium**	
Volume, min	3.4 ± 1 μL
Volume, notch	5 ± 1.3 μL
Volume, max	6.5 ± 1.5 μL
Early reservoir volume increase	1.6 ± 0.6 μL
Late reservoir volume increase	1.3 ± 0.5 μL
Total reservoir volume increase	3 ± 0.71 μL
MLD, min	1.7 ± 0.2 mm
SID, min	1.8 ± 0.2 mm
MLD, max	2.1 ± 0.1 mm
SID, max	2.3 ± 0.2 mm
**Left Atrial Appendage**	
LAA long axis, maximum	4 ± 0.2 mm
Duct diameter, minimum	0.89 ± 0.1 mm
Duct diameter, maximum	1.33 ± 0.05 mm
Duct diameter, fractional shortening	33 ± 10%
LAA stroke volume	10.6 ± 2.5 μL
**Right Superior Pulmonary Vein Flow Profile**	
Early systolic wave, peak velocity	250 ± 50 mm/s
Early systolic wave, integral	6 ± 1.4 mm
Late systolic wave, peak velocity	173 ± 50 mm/s
Late systolic wave, integral	3.2 ± 0.6 mm
Diastolic wave, peak velocity	681 ± 155 mm/s
Diastolic wave, integral	21 ± 6.8 mm
Total PV integral	30 ± 7.9 mm

MLD: medio-lateral diameter, SID: supero-inferior diameter, PV: pulmonary vein.

Values are mean±SD.

### Effects of LV Ischemia

**Fig 9:**
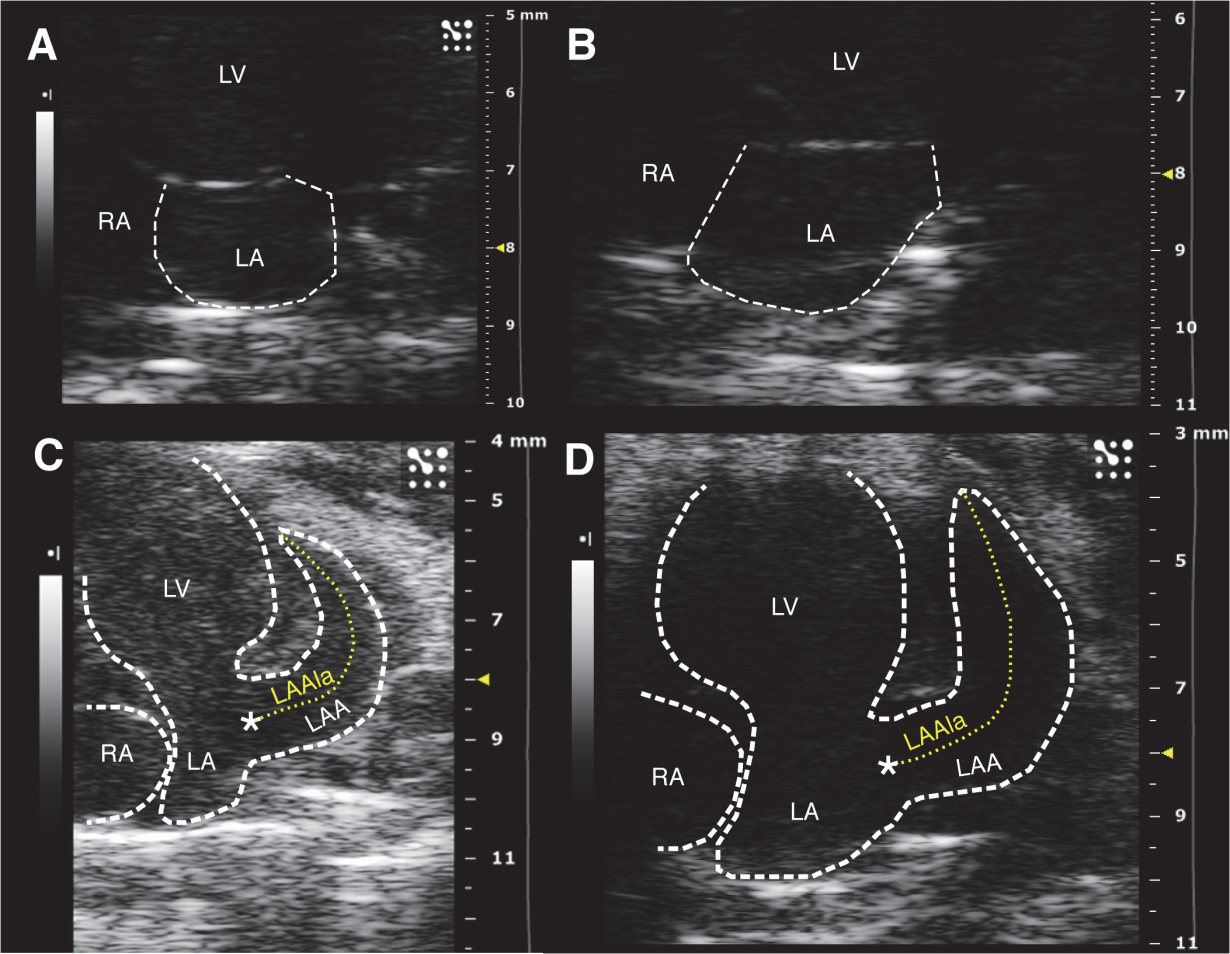
Left atrium and appendage changes following myocardial infarction. LA maximum area in normal (A) and 4-weeks ischemic mice (B). LAA long axis in normal (C) and ischemic mice (D). LA: left atrium; LAA: left atrial appendage; LAAla: LAA long axis; LV: left ventricle; RA: right atrium

No significant difference was found in HR compare to baseline (504±24 *vs* 519±34 bpm). Compare to sham group the LV EF decreased significantly confirming systolic impairment. LA volumes and LAA long axis length increased significantly after 1 week to became prominent at 4 weeks post-MI while the LA-LAA duct fractional shortening gradually decreased showing reduced duct contractility. The LAA SV as well as LV SV were both unchanged. Data of stuctural and functional modification occurring at 1 and 4—weeks after MI induction data are shown in Table 8 and in Fig 9.

**Table 8:** Myocardial Infarction.

week		LV EF (%)	LV SV (μL)	LA Vmax (μL)	LA Vmin (μL)	LA Vres (μL)	LA-LAA duct FS (μL)	LAA length (mm)	LAA SV (μL)
0	baseline	72.2 ± 7	36.2 ± 9	5.3 ± 1.7	2.3 ± 0.7	3.0 ± 1	49.6 ± 3	4.0 ± 0.35	11.4 ± 2.3
1	sham	73 ± 3	32.3 ± 4	5.2 ± 0.8	2.45 ± 1.5	2.75 ± 0.6	48.7 ± 5	3.8 ± 0.2	5.2 ± 2.4
	MI	36 ± 8**	25 ± 3.3	8 ± 2.8	4.2 ± 0.2**	3.8 ± 1.3**	35 ± 8	3.88 ± 0.4	5.8 ± 2.4
4	sham	72 ± 2.5	32 ± 3.3	5.5 ± 1.5	2.65 ± 0.9	2.85 ± 0.5	50.5 ± 12	3.85 ± 0.2	7.2 ± 2.5
	MI	30.0 ± 10**	35.2 ± 7	10.8 ± 4*	6.5 ± 3.5**	4.3 ± 1.2**	23.6 ± 10**	4.7 ± 0.35**	8.5 ± 3.5

EF: ejection fraction, FS: fractional shortening, LA: left atrium, LAA: left atrium appendage, LV: left ventricle, SV: stroke volume, Vmax: maximum volume, Vmin: minimum volume, Vres: volume reservoir. Values are mean±SD.

*p<0.05,

**p<0.01 relative to sham.

### Variability of Echocardiographic Measurements

The interaobserver variability within session was ≤ 9.6% and the intraobserver variability was ≤ 18%. Intersession variability within two session was ≤ 20%. Data are summarized in Table 9.

**Table 9:** Intra- and Intra-observer and intersession variability.

Index	Within session Error (%)		Intersession Error (%)
	Intraobserver	Interobserver	
LV stroke volume	5.6±4	6.7±5	15±8
LV base descent	4.6±4	4.5±4	13±7
LA volume, minimum	4.9±4	16.6±9	19.7±12
LA volume, notch	6.4±5	15.8±6	15.9±11
LA volume, maximum	5.2±3	12.3±8	11.1±8
LA volume, reservoir	9.1±6	11.9±7	11.3±8
LAA systolic integral	4.3±4	13.1±5	17.9±13
LAA diastolic integral	7.7±6	14.3±11	20±14
LA-LAA duct, minimum	8.3±6	7.4±6	7.2±6
LA-LAA duct, maximum	2.5±2	2.4±2	5.4±4
LAA stroke volume	9.6±8	18±10	19.7±12
PV systolic integral	7.9±7	14.3±11	13.8±10
PV total integral	4.9±4	5.7±5	14.6±9

LA: left atrium, LAA: left atrium appendage, LV: left ventricle, PV pulmonary vein. Values are mean±SD.

## Discussion

Structural similarities and the availability of transgenic lines make the mouse a powerful model to identify the mechanisms underlying human cardiovascular development, function and disease. Strong evidence suggest that the left atrial enlargement can be used as clinical indicator of significant risk of adverse cardiovascular outcomes for the patient, however to investigate the possibility to use LA size as prognostic value, an understanding of the LA structure and function in animal models and its association with cardiovascular disease is required. This study present a systematic characterization of the murine left venous reservoir providing macroscopic anatomy together with histologic and echocardiographic reference values of its main components: LA, LAA and PVs. Our echocardiographic approch and macroscopic findings were also verified on the outbred CD-1 female mice and on the inbred FVB male mice. The echocardiographic protocol was also tested on a model of LV ischemia to evaluate its usefulness in evaluating acute and sub-acute stress.

The murine atrium is disproportionally small compare to LV, according to our data LA to LV volume ratio is 0.1 compare to 0.58 reported in humans [14]. The atrium is connected through a contractile duct to a much larger appendage, which provides a flow volume four times the LA reservoir volume, enhancing reservoir function to support LV SV. The LA volume reservoir alone accounts for only 7–8% of LV SV while LAA SV together with LA volume reservoir account for about 36% of LV SV and the rest is provided by conduit flow.

The cellular composition of LA and LAA is similar, with predominant cardiomyocyte component confirming the role of LAA as a contractile chamber working in synergy with LA. Therefore atrium and appendage should be considered almost as a single chamber suggesting that, in mouse, the large appendage plays a central role compare to its negligible role in humans, in which LAA can be closed to prevent thrombosis risk [15]. To further support this hypothesis we observe that the LAA SV is the main independent determinant of LV SV.

The 2D apical 4-chamber view allowed to calculate LA volumes, area function curve as in other species [3, 16] and to characterize anatomical details. Area function curve shows that, in mice, early LA filling phase (up to the notch) is prevalent in both duration and volume increase, contrasting with the prevalently late reservoir filling in humans [17]. This evidence suggests that, in mice, reservoir filling is dominated by relaxation, since the main determinants of LA early and late filling phases are respectively LA relaxation and compliance [3, 11, 17–20]. The prevalent role of relaxation is supported also by the strong correlation between the LA early filling volume and LV SV found at univariate linear regression.

Macroscopic anatomy and color Doppler imaging show that LA is supplied by three pulmonary veins (PVs). Few histological studies describe in mice a single PV entering the LA chamber dorsally [21, 22]; in our histological experiments we were unable to describe the anatomy and clarify the numbers of PVs. Another study, that combined *in vivo* echocardiography and corpse magnetic resonance imaging, hypothesized a single PV [23]; however their echocardiographic equipment was less powerful with respect to both temporal and spatial resolution, also on corpse some PVs were probably hidden by their collapse after death. Evidence on C57BL/6N mice were also confirmed by analises on other mouse strain such as CD-1 and FVB mice.

In this experiment the full set of LV parameters analyzed were comparable with those already reported [23, 24]. A single mitral flow wave (following ECG P wave) was observed implying the synchronous occurrence of elastic recoil [3, 25], conduit, and contraction during LV relaxation [26], nevertheless in agreement with other studies [27] the typical E and A waves pattern could be visualized by slowing down the HR by increasing the anesthesia confirming that the use of MV flow to evaluate diastolic function is highly influenced by HR and loading condition [8].

Many studies reported echocardiography as a feasible and valid method to evaluate LV size and function in normal adult mice and in several mouse models of human disease, such as myocardial ischemia [28], cardiomyopathy [29], hypertrophy [30]. As, to our knowledge, this is the first effort made to estimate LA and LAA size and function we also report here the ability of our echocardiographic approach to discriminate changes on LA and LAA after acute stress using a model of LV ischemia. Our results show that echocardiography is of great potential to noninvasively assess effects and compare differences in murine models of stress, information that could be used for future pharmacological, cellular or tissue engineering studies.

## Conclusions

We describe a high-frequency ultrasound imaging protocol for the left venous reservoir that allows a comprehensive assessment of the C57BL/6N mouse cardiovascular phenotype. In contrast with humans, mice have a large and functionally important LAA, a disproportionately small LA, and three PVs entering the LA. Atrial relaxation is an important determinant of LV stroke volume variation, although most of LV filling is provided by atrial conduit function at the high heart rates observed in mice.

Mitral valve flow, commonly used to evaluate LV diastolic function, is sensitive to heart rate and loading conditions; therefore we suggest the LA, LAA and PV function variables as a potential alternative. However further studies are needed to validate and develop our findings possibly supported by advances in imaging technology with improved spatial and temporal resolution.
